# Supplementary material for: Diesel soot photooxidation enhances the heterogeneous formation of H2SO4
Source: Nat Commun. 2022 Sep 12;13:5364. doi: 10.1038/s41467-022-33120-3 (PMC9467980; doi:10.1038/s41467-022-33120-3)
Supplement: Supplementary file 1 — Supplementary Information [file 41467_2022_33120_MOESM1_ESM.pdf]

Supporting Information

Diesel soot photooxidation enhances the heterogeneous formation of H_2SO_4

Peng Zhang [1], Tianzeng Chen [1,2], Qingxin Ma [1, 2, 3]*, Biwu Chu[1, 2, 3], Yonghong Wang [1,2], Yujing Mu [1, 2, 3], Yunbo Yu [1, 2, 3], and Hong He [1, 2, 3]*

[1] State Key Joint Laboratory of Environment Simulation and Pollution Control, Research Center for Eco-Environmental Sciences, Chinese Academy of Sciences, Beijing 100085, China

[2] University of Chinese Academy of Sciences, Beijing 100049, China

[3] Center for Excellence in Regional Atmospheric Environment, Institute of Urban Environment, Chinese Academy of Sciences, Xiamen 361021, China

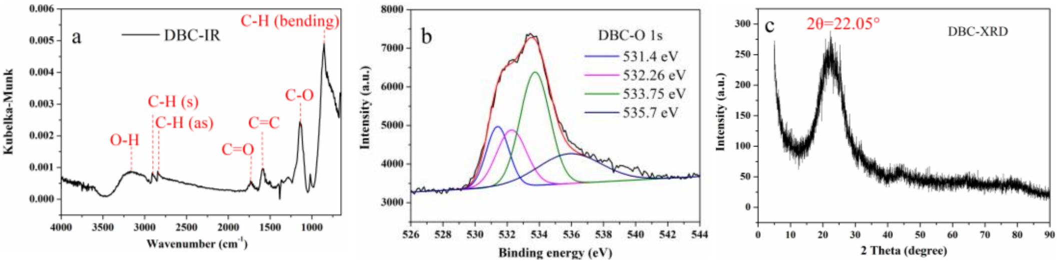

Supplementary Fig. S1 DBC characterization. *in situ* DRIFTS (A); High-resolution XPS spectrum of O 1s (B); XRD pattern (C).

The DRIFTS spectrum of DBC was shown in Fig.S1a. The main peaks in the range of 3000-3500 cm^{-1} represent the stretching vibrations of H-O bonds in adsorbed H_2O molecule or alcohol or phenol species. The peaks from 1051 to 1238 cm^{-1} represent the stretching vibration of C-O bonds in epoxy/ether and phenol/alcohol group.[1, 2] The peak at 1726 cm^{-1} was the characterize vibration of C=O bonds in CO(O)H group.[1] The vibration peak at 1587 cm^{-1} was ascribed to the C=C bonds in graphite or graphene lattice.[1, 3, 4] Both of peaks at 2933 and 2864 cm^{-1} represent the symmetry and asymmetry stretching vibration of C-H bonds in alkanes. The strong vibration at 860 cm^{-1} were mainly assigned to be bending vibration of C-H bonds. The high-resolution O 1s spectrum in Fig.S1b further supported above results. The O1s bonds of DBC can be deconvoluted into four peaks, relevant to oxygen doubly bond to carbon (O=C-O) in ester or carboxylic acids (531.4 eV), carbon-oxygen single bond (C-O) in phenol, alcohol, and ether functions (532.26 eV), oxygen of aryl ethers (533.75 eV), and chemisorbed oxygen or water (535.7 eV).[5-8] In Fig. S1c, the XRD pattern of DBC is characteristic of graphite with a layer spacing identical to that of bulk graphite (3.34 Å).[9] Together with the observed C=C stretch vibration of graphite lattice (1587 cm^{-1} in Fig. S1a), these results indicated that DBC was amorphous forms and high graphitization.

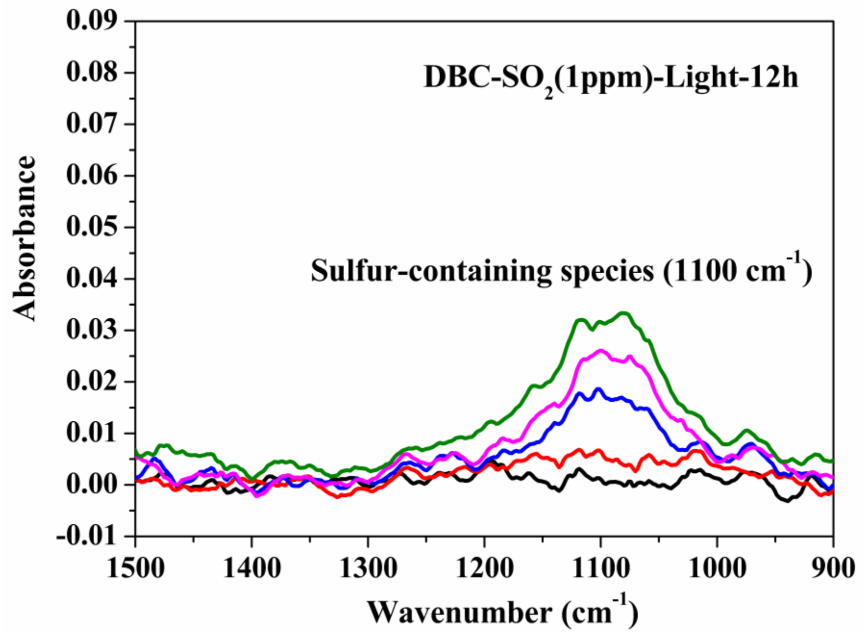

Supplementary Fig. S2 In situ DRIFTS spectra of sulfur-containing species. DBC was exposed to 1 ppm SO_2 for 12 h under light irradiation.

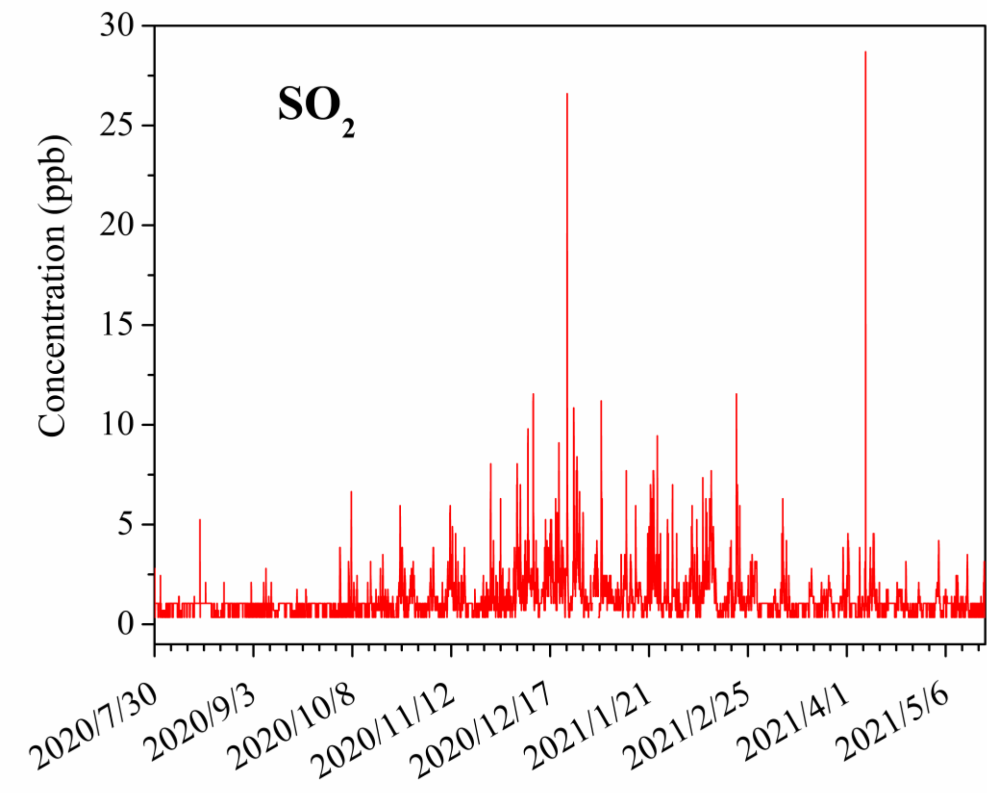

Supplementary Fig. S3 Field observation of SO_2. The hourly average concentration of SO_2 from 30 July 2020 to 20 May 2021 in Beijing.

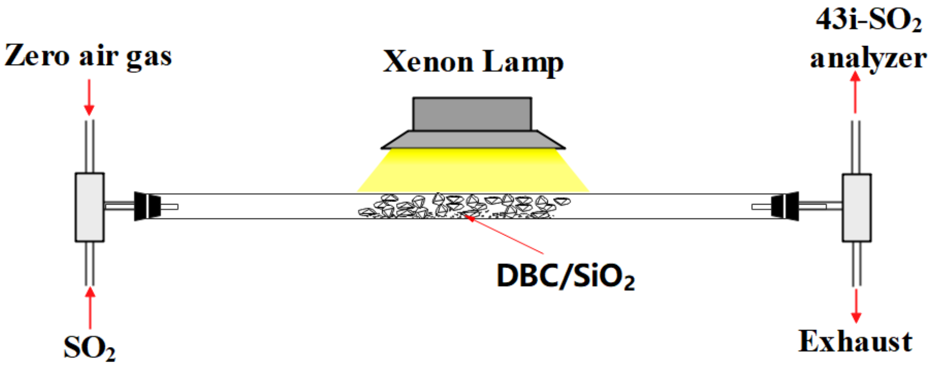

Supplementary Fig. S4 The schematic diagram. Tube plug flow reactor setup.

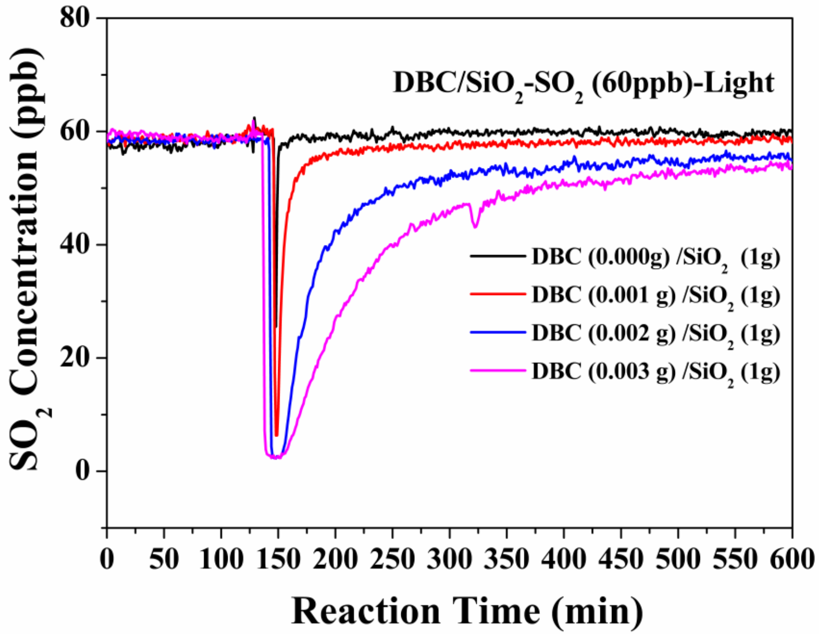

Supplementary Fig. S5 SO_2 uptake. The uptake of SO_2 on DBC with different mass concentrations.

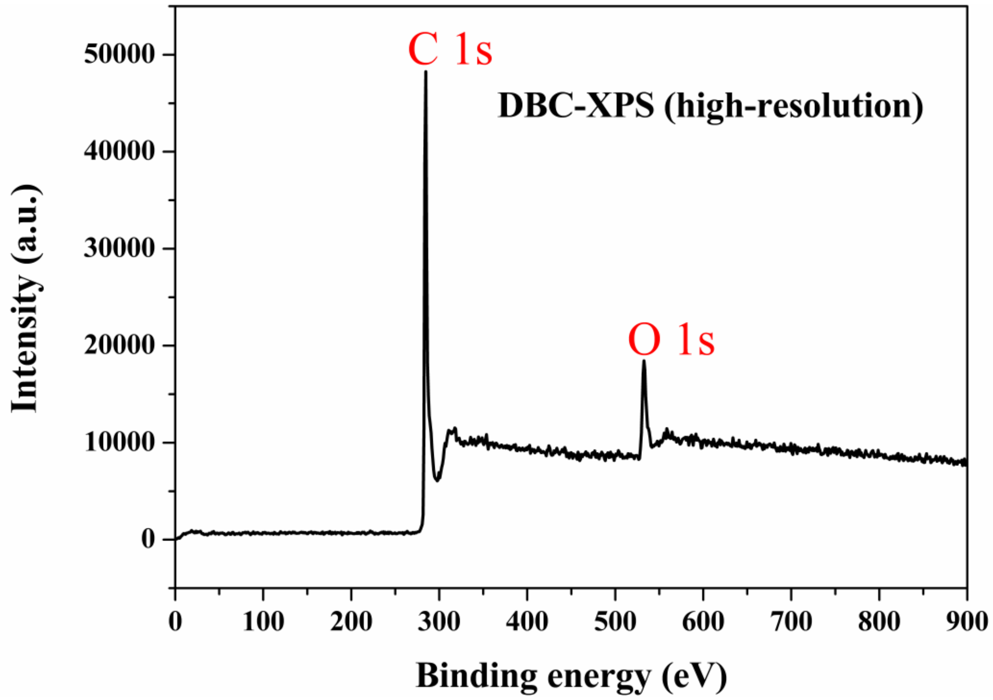

Supplementary Fig. S6 XPS analysis of DBC. Survey XPS spectrum of fresh DBC

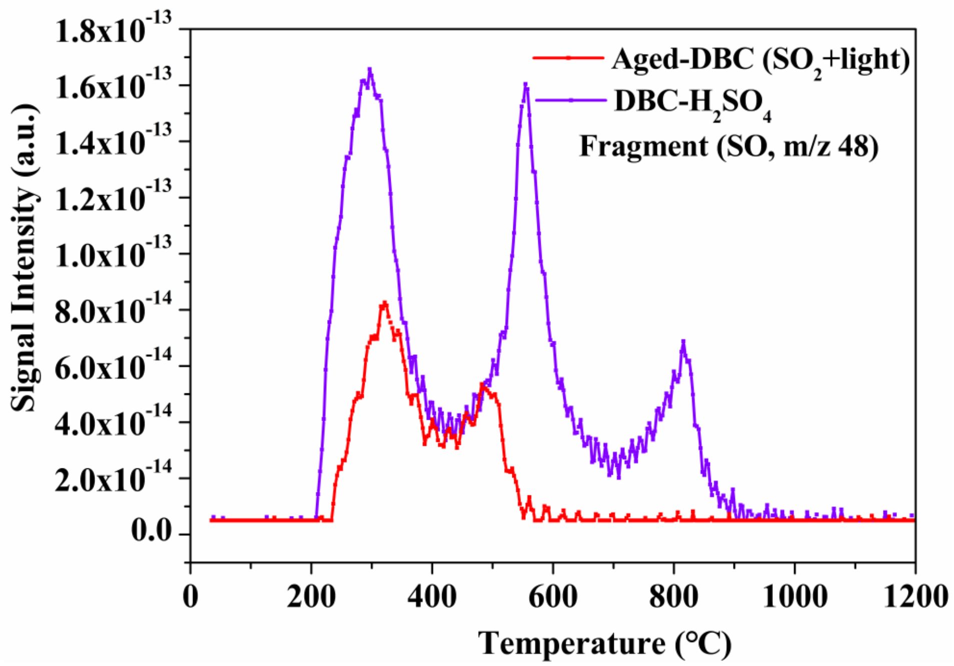

Supplementary Fig. S7 SO fragment (m/z 48). The evolution of SO fragment during the thermal desorption of aged-DBC by TAG-MS.

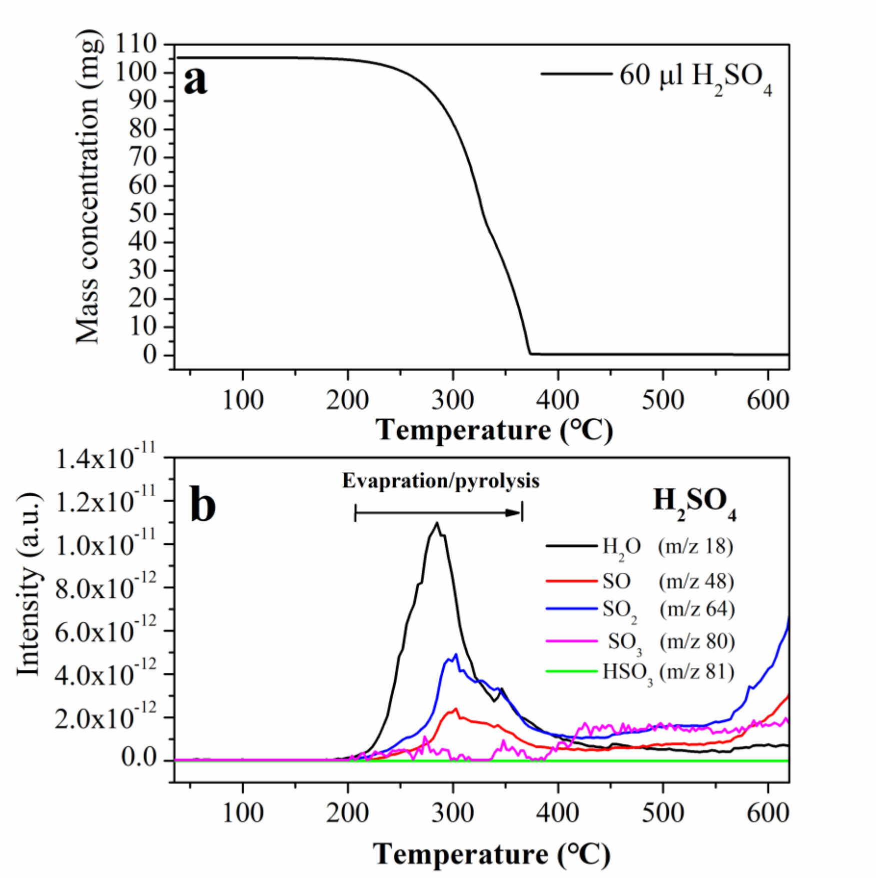

Supplementary Fig. S8 TGA-MS analyzation of pure H_2SO_4. The evolution of sulfur-containing fragment during the TGA-MS of pure H_2SO_4: (a) TGA curve; (b) Mass spectrum analyzation.

A new TGA-MS experimental result of ~60 μL pure H_2SO_4 (Fig.S8a) showed that the major weight loss happens in the temperature range of 200 ℃ to 400 ℃. Thus, the evaporation and decomposition of H_2SO_4 takes place simultaneously in this temperature range, which can be supported by the observation of both H_2O (m/z 18) and SO_3 (m/z 80) (Fig. S8b). Unlike the MS spectrum of H_2SO_4, m/z=80 is not the main fragment of SO_3. Meanwhile, the sulfur-containing fragments such as SO_2 (m/z 64) and SO (m/z 48) mainly originates from the further fragmentation of SO_3 during the electronic ionization.[10] Here, both SO and SO_2 fragments are chosen to trace the formation of H_2SO_4 due to the high signal intensity in TGA-MS analysis.

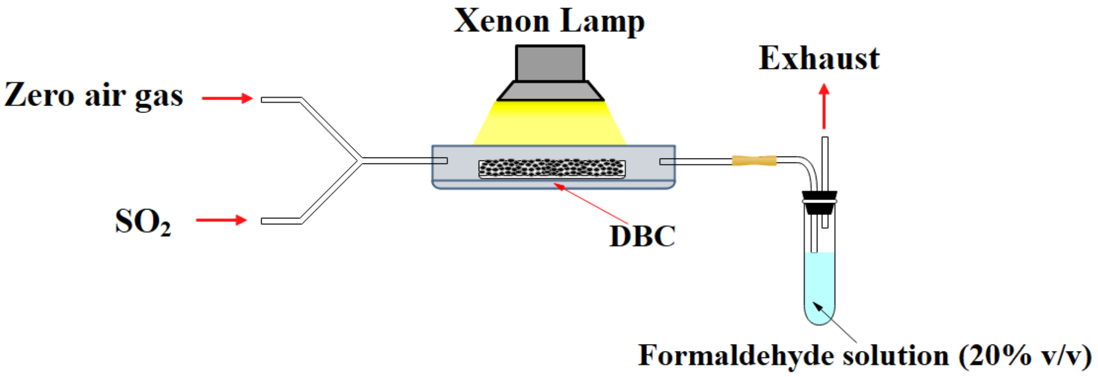

Supplementary Fig. S9 The schematic diagram. The quartz photoreaction flow tank.

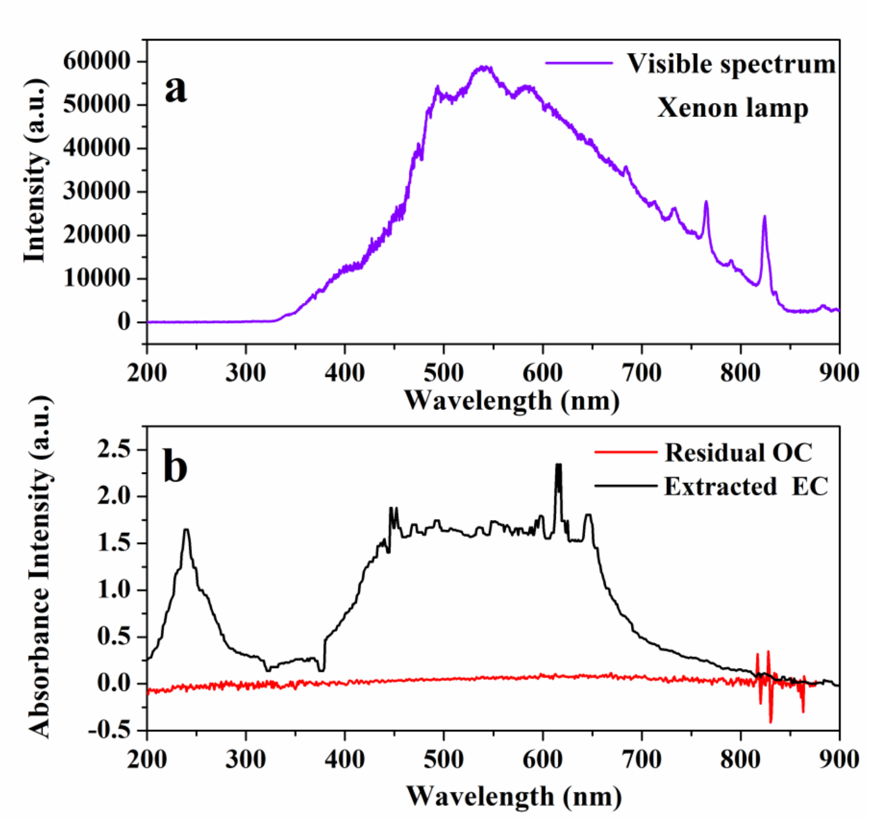

Supplementary Fig. S10 The visible spectrum and UV-vis spectra. The visible spectrum of Xenon light (a); The UV-vis spectra of residual EC and extracted OC (b).

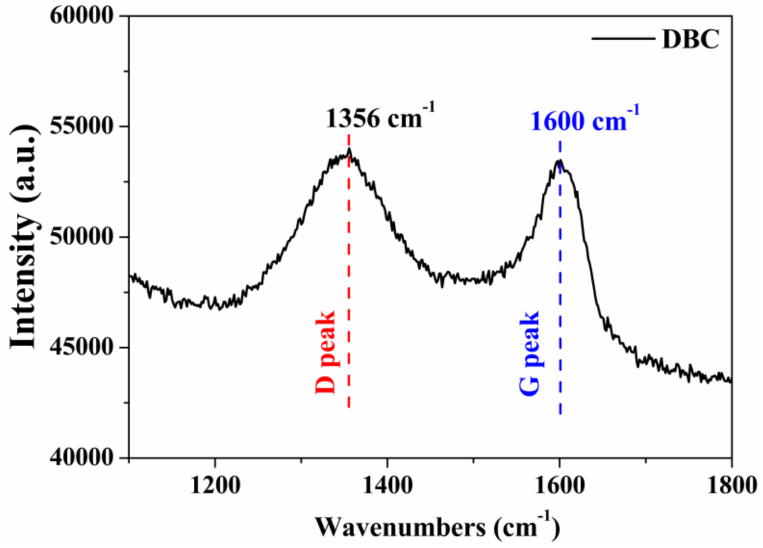

Supplementary Fig. S11 Raman analyzation of DBC. The Raman spectra of DBC sample.

In Fig. S11, the Raman spectra of DBC samples showed two major characteristic peaks: the D peak (~1356 cm^{-1}) representing the local defects (or *sp*^{3} bonding) or disorder and the G peak (~1600 cm^{-1}) originating from the graphene *sp*^{2} network. This indicates that the structure defects or disorder structures are also ubiquitous in DBC.

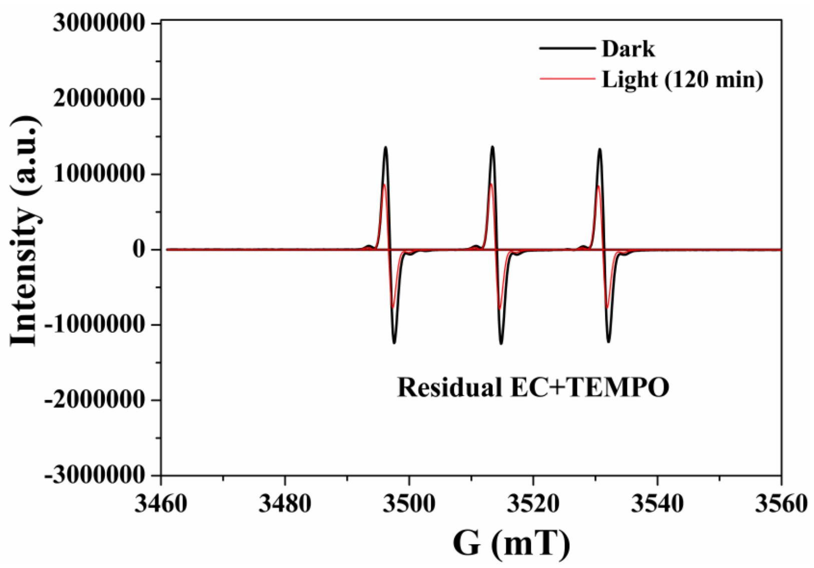

Supplementary Fig. S12 ESR characterization of photoinduced electrons. TEMPO spin-trapping ESR spectra for the detection of electrons in residual EC suspension

Supplementary Reference

1. Alrefaai MM, Pena GDJG, Raj A, Stephen S, Anjana T, Dindi A. Impact of dicyclopentadiene addition to diesel on cetane number, sooting propensity, and soot characteristics. *Fuel.* **216**, 110-120 (2018).
2. Antinolo M, Willis MD, Zhou SM, Abbatt JPD. Connecting the oxidation of soot to its redox cycling abilities. *Nat. Commun.* **6**, (2015).
3. Wang L, *et al.* Gram-scale synthesis of single-crystalline graphene quantum dots with superior optical properties. *Nat. Commun.* **5**, (2014).
4. Wang L, *et al.* Full-color fluorescent carbon quantum dots. *Sci. Adv.* **6**, (2020).
5. Fan LZ, Liu JL, Ud-Din R, Yan XQ, Qu XH. The effect of reduction time on the surface functional groups and supercapacitive performance of graphene nanosheets. *Carbon.* **50**, 3724-3730 (2012).
6. Chen XX, Chen BL. Macroscopic and Spectroscopic Investigations of the Adsorption of Nitroaromatic Compounds on Graphene Oxide, Reduced Graphene Oxide, and Graphene Nanosheets. *Environ. Sci. Technol.* **49**, 6181-6189 (2015).
7. Niessner R. The Many Faces of Soot: Characterization of Soot Nanoparticles Produced by Engines. *Angew. Chem. Int. Edit.* **53**, 12366-12379 (2014).
8. Frank B, Schuster ME, Schlogl R, Su DS. Emission of Highly Activated Soot Particulate - The Other Side of the Coin with Modern Diesel Engines. *Angew. Chem. Int. Edit.* **52**, 2673-2677 (2013).
9. Stobinski L, *et al.* Graphene oxide and reduced graphene oxide studied by the XRD, TEM and electron spectroscopy methods. *J. Electron. Spectrosc.* **195**, 145-154 (2014).
10. Smith OI, Stevenson JS. Determination of Cross-Sections for Formation of Parent and Fragment Ions by Electron-Impact from SO_2 and SO_3. *J. Chem. Phys.* **74**, 6777-6783 (1981).
